# Prognostic value of CBV index in patients with acute ischemic stroke treated with endovascular thrombectomy in late therapeutic window

**DOI:** 10.3389/fneur.2023.1282159

**Published:** 2024-01-08

**Authors:** Aicheng Sun, Yuezhou Cao, Zhenyu Jia, Linbo Zhao, Haibin Shi, Sheng Liu

**Affiliations:** The First Affiliated Hospital of Nanjing Medical University, Nanjing, China

**Keywords:** CBV index, acute ischemic stroke, collateral circulation, endovascular thrombectomy, intravenous thrombolysis, large vessel occlusion

## Abstract

**Purpose:**

To evaluate the prognostic value of the cerebral blood volume (CBV) index for 90-day functional outcomes in patients with acute ischemic stroke (AIS) treated within a late therapeutic window.

**Methods:**

We retrospectively reviewed patients who underwent pre-treatment computed tomography perfusion (CTP) and endovascular thrombectomy (EVT) for large-vessel occlusion (LVO) of the anterior circulation within the late therapeutic window between January 2021 and February 2023. Clinical data, the Alberta Stroke Program Early Computed Tomography Score (ASPECTS) based on unenhanced computed tomography (CT), and perfusion parameters, including ischemic core, hypoperfusion volume, mismatch volume between the core and penumbra, and CBV index, were assessed and compared between patients who achieved favorable outcomes (defined as a modified Rankin Scale score of 0–2).

**Results:**

Of the 118 patients, 56 (47.5%) had favorable outcomes. In the univariate analysis, age, National Institutes of Health Stroke Scale (NIHSS) score at admission, ASPECTS score, CBV index, and ischemic core volume were significantly associated with functional outcomes (*P* < 0.05). In multivariate analyses, age (odds ratio [OR], 1.060; 95% confidence interval [CI] 1.013–1.110, *P* = 0.012), NIHSS score at admission (OR, 1.126; 95% CI 1.031–1.229, *P* = 0.009), and CBV index (OR, 0.001; 95% CI 0.000–0.240, *P* = 0.014) were independent predictors of a 90-day favorable outcome.

**Conclusion:**

A high CBV index was independently associated with favorable outcomes in patients who underwent mechanical thrombectomy within the late therapeutic window. In addition, a higher CBV index reflects improved blood flow and favorable digital subtraction angiography collateral status.

## 1 Introduction

Recently, several randomized controlled trials have provided evidence supporting the efficacy and safety of endovascular thrombectomy (EVT) for the treatment of stroke due to large-vessel occlusion (LVO) in the anterior circulation (1). At present, the therapeutic window for stroke treatment has been extended to 24 h (2). However, despite strict eligibility criteria based on neuroimaging or a combination of National Institutes of Health Stroke Scale (NIHSS) scores for patient selection, the overall functional outcome indicated by modified Rankin Scores (mRS) of 0–2 at 90 days in these treatment groups was <50% (1). The clinical outcomes of patients treated within the late therapeutic window may be more difficult because of the longer ischemic time as opposed to patients treated within the early therapeutic window.

Although some predictors can be used to predict treatment outcomes, the presence of favorable collateral circulation is still considered one of the most reliable predictors of favorable long-term clinical outcomes. Computed tomography angiography (CTA) is frequently used for collateral assessment; however, the DEFUSE 3 study revealed that the collateral score derived from CTA could not accurately predict outcomes in patients treated in the late therapeutic window (3). Computed tomography perfusion (CTP) is currently recommended for clinical decision-making in patients with acute ischemic stroke (AIS). Some traditional CTP parameters including volume of ischaemic core and penumbra have been used to select AIS patients eligible for EVT, but yield discrepancy performance for outcome prediction (4, 5). The cerebral blood volume (CBV) index is derived from perfusion imaging and is defined as the average CBV in the Tmax > 6 s region compared with the average CBV in normal brain tissue. Subsequently, one study reported that the CBV index is related to the collateral status and could predict infarct growth after EVT (6, 7). However, this parameter has not been assessed in patients presenting with LVO over extended time windows.

The present study aimed to investigate the performance of the CBV index compared with traditional CTP parameters as an imaging biomarker to predict 90-day functional outcomes in patients with AIS who underwent EVT within the late therapeutic window.

## 2 Materials and methods

### 2.1 Patients

Patients presenting with LVOs within the anterior circulation on CTA who underwent EVT between January 2021 and February 2023 were retrospectively analyzed. The inclusion criteria for the study were as follows: (a) stroke onset time to groin puncture ≥6 h, or last witnessed normal to groin puncture ≥6 h; (b) AIS with occlusion of an anterior circulation artery (intracranial internal carotid artery [ICA], middle cerebral artery segment 1 [M1] and/or middle cerebral artery segment 2 [M2]) confirmed by CTA; (c) age >18 years old; (d) those who met the eligibility criteria of DEFUSE 3, including an ischemic core volume <70 ml, mismatch ratio 1.8, and mismatch volume >15 ml; (e) National Institutes of Health Stroke Scale (NIHSS) scores >6 on admission; and (f) the image quality of CTP was good without significant motion artifacts. The exclusion criteria were as follows: (a) incomplete clinical and imaging data; (b) baseline modified Rankin Scale (mRS) scores >2; and (d) previous intracranial hemorrhage.

### 2.2 Clinical information

Clinical data were collected from the stroke database of the stroke center, including demographic information (age and sex), stroke risk (hypertension, diabetes, coronary artery disease, atrial fibrillation, smoking, and history of ischemic stroke), and relevant information on stroke (National Institutes of NIHSS score at admission, use of intravenous thrombolysis [recombinant tissue plasminogen activator (rt-PA)], and stroke onset-to-groin puncture time). Functional outcomes were assessed at 90 days using the mRS. During face-to-face visits or structured telephone interviews, board-certified stroke neurologists prospectively evaluated the mRS score. These assessments were carried out while the neurologists blinded to all imaging or clinical details. A favorable functional outcome was defined as an mRS of 0–2 at 90 days.

Non-contrast computed tomography (NCCT), CTA, and CTP scans were performed using a 256-slice multidetector computed tomography (CT) scanner (Optima CT 660; GE Medical Systems, Chicago, IL, USA) according to our institutional stroke imaging protocol for patients with AIS. CTP was post-processed using rapid processing of Perfusion and Diffusion (RAPID) software (iSchemaView, Menlo Park, California, USA). The CTP parameters derived from RAPID included the Alberta stroke program early CT score (ASPECTS), cerebral blood flow (CBF), cerebral blood volume (CBV), and time to maximum residue function (Tmax). The volume of the infarction core was defined as the volume of brain tissue with CBF <30%. Parametric maps were generated automatically. The CBV index was defined as the average CBV in the Tmax >6 s region compared to the average CBV in normal brain tissue (6).

On the ASITN scale, we classified levels 0 and 1 as poor, level 2 as fair, and levels 3 and 4 as good collaterals for DSA. Successful angiographic reperfusion was defined as a modified Thrombolysis in Cerebral Infarction (mTICI) score of 2b or 3. A favorable outcome at 90 days was defined as a modified Rankin Scale score of 0–2.

### 2.3 Statistical analysis

The χ2 test or Fisher exact test was used to compare categorical clinical and imaging characteristics and the Mann Whitney U test was used to compare continuous variables. We used ANOVA statistic to compare three different groups. Differences were considered statistically significant at *P* < 0.05. All statistical analyses were performed using IBM SPSS (version 20.0; IBM).

## 3 Results

Between January 2021 and February 2023, 118 patients with AIS who were admitted within the late therapeutic window and met all inclusion criteria were enrolled. The median age of these patients was 71 years (interquartile range [IQR], presented as the 25th and 75th percentiles, 62–78), and 35.6% were women. These patients had occlusions of the ICA (*n* = 31), MCA-M1 (*n* = 81), and MCA-M2 (*n* = 6) segments. The median NIHSS score at admission was 15 (IQR, 10–20). The stroke onset-to-groin puncture time ranged from 362 to 1,406 min (median, 549, IQR 422–742). On the CTP parameter maps, the median lesion volume of the ischemic core and the hypoperfusion volume defined by Tmax >6 were 13 ml (IQR, 0–27), and 145 ml (IQR, 95–186), respectively. The median CBV index was 0.7 (IQR, 0.6–0.8). Favorable outcomes were achieved in 56 (47.5%) patients after 90 days.

Table 1 shows a comparison of the demographic and clinical factors in patients with good, fair, and poor DSA collateral statuses. Overall, there were no significant differences in sex among the three groups. Significant differences were observed across the three groups (good, fair, and poor) for age (63.2, 70.4, 72.2, *P* = 0.001), baseline NIHSS score (11.0, 17.0, 20.0, *P* < 0.001), atrial fibrillation (34.0%, 52.4%, 63.8%, *P* = 0.013), baseline core infarct volume (3.0, 11.0, 27.0, *P* < 0.001), baseline ASPECTS score (8.0, 7.0, 5.0, *P* < 0.001), final mTICI score of 2b+ (94.0%, 90.4%, 74.5%, *P* = 0.018), 90-day mRS of 0 to 2 (78.0%, 61.9%, 19.1%, *P* < 0.001), and CBV index (0.8, 0.7, 0.6, *P* < 0.001). The specifications of the pairwise contrasts are listed in Table 2.

**Table 1:** Comparison of demographic and clinical end points in subjects with good, fair, and poor collaterals.

**Variables**	**Good collaterals (*n =* 50)**	**Fair collaterals (*n =* 21)**	**poor collaterals (*n =* 47)**	***P*-value**
Patient data				
Age, years, median (IQR)	63 (51–75)	69 (60–81)	73 (65–87)	0.001
Female, *n* (%)	17 (36.2%)	10(47.6%)	15(31.9%)	0.437
History				
Hypertension, *n* (%)	29(58.0%)	13(61.9%)	32(68.1%)	0.588
Diabetes mellitus, *n* (%)	5(10.0%)	2(9.5%)	2(4.3%)	0.353
Hyperlipidemia, *n* (%)	3(6.0%)	2(9.5%)	6(12.8%)	0.519
Atrial fibrillation, *n* (%)	17(34.0%)	11(52.4%)	30(63.8%)	0.013
Prior stroke, *n* (%)	2(4.0%)	2(9.5%)	4(2.1%)	0.243
Smoking, *n* (%)	9(18.0%)	0(0%)	5(10.6%)	0.096
Coronary heart disease, *n* (%)	9(18.0%)	2(9.5%)	2(4.3%)	0.385
Baseline NIHSS, median (IQR)	11 (9–15)	17 (10–21)	20 (14–22)	< 0.001
Baseline ASPECTS, median (IQR)	8 (7–8)	7 (6–8)	5 (4–7)	< 0.001
Occlusion site				0.185
ICA, *n* (%)	12 (24.0%)	2 (9.5%)	17 (36.2%)	
MCA-M1, *n* (%)	36 (72.0%)	18 (85.7%)	27 (57.4%)	
MCA-M2, *n* (%)	2 (4.0%)	1 (4.8%)	3 (6.4%)	
Etiology of the occlusion				0.370
Cardioembolism, *n* (%)	19 (38.0%)	11 (52.4%)	27 (57.4%)	
Large artery atherosclerosis, *n* (%)	26 (52.0%)	9 (42.9%)	16 (34.0%)	
Others, *n* (%)	5 (10.0%)	1 (4.8%)	4 (8.5%)	
IV tPA, *n* (%)	14 (28.0%)	3 (14.3%)	9 (19.1%)	0.368
Onset to puncture, median (IQR)	542 (462–784)	585 (440–798)	550 (410–692)	0.759
Onset to Reperfusion, median (IQR)	633(550–858)	639 (531–836)	635 (488–764)	0.691
CTP parameters				
CBF < 30% volume in ml, median (IQR)	3 (0–16)	11 (5–17)	27 (13–44)	< 0.001
Mismatch volume (mL), median (IQR)	129 (79–154)	115 (70–146)	132 (96–175)	0.337
Tmax >6 seconds, median (IQR)	134 (85–175)	131 (87–171)	166 (122–203)	0.023
CBV index, median (IQR)	0.8 (0.8–0.9)	0.7 (0.7–0.8)	0.6 (0.6–0.7)	< 0.001
mTICI 2b/3, *n* (%)	47(94.0%)	19 (90.4%)	35 (74.5%)	0.018
90-day good outcome (mRS score 0–2)	39 (78.0%)	13 (61.9%)	9 (19.1%)	< 0.001

ICA, internal carotid artery; MCA, middle cerebral artery; NIHSS, National Institutes of Health Stroke Scale score; ASPECTS, Alberta Stroke Program Early Computed Tomography Score; CBF, cerebral blood flow; CBV, cerebral blood volume; Tmax, time to maximum; rt-PA, recombinant tissue plasminogen activator; mTICI, modified Treatment in Cerebral Ischemia. Data are expressed as median and interquartile range (IQR) (expressed as the 25th percentile. and the 75th percentiles, respectively).

**Table 2:** Comparison of clinical variables and neuroimaging characteristics in patients with favorable vs. unfavorable outcomes at 90 days.

**Variables**	**Favorable outcome (*n =* 56)**	**Unfavorable outcome (*n =* 62)**	***P*-Value**
Patient data			
Age, years, median (IQR)	65 (52–78)	74 (63–86)	< 0.001
Female, *n* (%)	18 (32.1%)	24 (38.7%)	0.581
History			
Hypertension, *n* (%)	33 (58.9%)	41 (66.1%)	0.537
Diabetes mellitus, *n* (%)	7 (12.5%)	9 (14.5%)	0.960
Hyperlipidemia, *n* (%)	4 (7.1%)	7 (11.3%)	0.648
Atrial fibrillation, *n* (%)	22 (39.3%)	36 (58.1%)	0.064
Prior stroke, *n* (%)	6 (10.7%)	2 (3.2%)	0.212
Smoking, *n* (%)	7 (12.5%)	7 (11.3%)	1.000
Coronary heart disease, *n* (%)	4 (7.1%)	6 (9.7%)	0.871
Baseline NIHSS, median (IQR)	11 (9–16)	20 (13–23)	< 0.001
Baseline ASPECTS, median (IQR)	8 (6–8)	6 (4–7)	0.001
Occlusion site			0.703
ICA, *n* (%)	16 (28.6%)	15 (24.2%)	
MCA-M1, *n* (%)	38 (67.9%)	43 (69.4%)	
MCA-M2, *n* (%)	2 (3.6%)	4 (6.5%)	
Etiology of the occlusion			0.203
Cardioembolism, *n* (%)	23 (41.1%)	34 (54.8%)	
Large artery atherosclerosis, *n* (%)	29 (51.8%)	22 (35.5%)	
Others, *n* (%)	4 (7.1%)	6 (9.7%)	
IV tPA, *n* (%)	14 (25.0%)	12 (19.4%)	0.606
Onset to puncture, median (IQR)	540 (416–684)	599 (448–792)	0.192
Onset to Reperfusion, median (IQR)	633 (499–746)	655 (548–859)	0.203
CTP parameters			
CBF < 30% volume in ml, median (IQR)	7 (0–18)	19 (7–40)	< 0.001
Mismatch volume (mL), median (IQR)	129 (84–160)	124 (84–158)	0.878
Tmax >6 seconds, median (IQR)	138 (86–177)	155 (102–192)	0.182
CBV index, median (IQR)	0.8 (0.7–0.9)	0.7 (0.6–0.7)	< 0.001
mTICI 2b/3, n (%)	50 (89.3%)	51 (82.3%)	0.410

ICA, internal carotid artery; MCA, middle cerebral artery; NIHSS, National Institutes of Health Stroke Scale score; ASPECTS, Alberta Stroke Program Early Computed Tomography Score; CBF, cerebral blood flow; CBV, cerebral blood volume; Tmax, time to maximum; rt-PA, recombinant tissue plasminogen activator; mTICI, modified Treatment in Cerebral Ischemia. Data are expressed as the median and interquartile range (IQR) (expressed as the 25th percentile and the 75th percentiles, respectively).

In the univariate analyses, patients with good functional outcomes were younger (*P* < 0.001), had lower NIHSS scores at admission (*P* < 0.001), had higher ASPECTS scores (*P* = 0.001), had smaller CBF perfusion deficits (*P* < 0.001), and had a higher CBV index (*P* < 0.001). There were no statistically significant differences between the two groups in terms of risk factors, stroke onset-to-groin puncture time, or vessel occlusion site. Detailed patient characteristics and univariate analyses of outcomes are shown in Table 2.

A multivariate logistic regression was performed using the statistically significant variables in univariate analysis as predictor variables and clinical outcomes as the dependent variable, including age, baseline NIHSS score, baseline ASPECTS, ischemic core volume and CBV index. Old age (odds ratio [OR], 1.060; 95% confidence interval [CI] 1.013–1.110, *P* = 0.012) and high NIHSS score at admission (OR, 1.126; 95% CI 1.031–1.229, *P* = 0.009) were independent predictors for a 90-day unfavorable outcome. Low CBV index (OR, 0.001; 95% CI 0.000–0.240, *P* = 0.014; Table 3) was independent predictors for a 90-day unfavorable outcome.

**Table 3:** Multivariable analyses for predicting a unfavorable outcome.

**Variables**	**OR (95% CI)**	***P*-Value**
Age	1.060 (1.013–1.1110)	0.012
Baseline NIHSS	1.126 (1.031–1.229)	0.009
Baseline ASPECTS	0.901 (0.680–1.192)	0.465
CBF < 30% volume in ml	1.016 (0.974–1.060)	0.460
CBV index	0.001 (0.000–0.240)	0.014

OR, odds ratio; CI, confidence interval; ASPECTS, Alberta Stroke Program Early Computed Tomography Score; CBF, cerebral blood flow; CBV, cerebral blood volume; Tmax, time to maximum; NIHSS, National Institutes of Health Stroke Scale.

## 4 Discussion

Forecasting the clinical outcomes of EVT in patients with AIS in the late therapeutic window remains challenging. The results of this study suggest that the CTP parameter, the CBV index, which can be calculated rapidly with automated software, could be a valuable marker for predicting the 90-day clinical outcome in AIS patients within the late therapeutic window after EVT. Our study indicated that a higher CBV index was associated with a favorable outcome, suggesting that the CBV index may be employed as a screening indicator. In addition, our study suggests that a higher CBV index reflects improved blood flow and favorable DSA collaterals.

A previous study indicated that the CBV index, defined as the relative CBV in the Tmax > 6 s region, is correlated with collateral circulation (6). The CBV index is a sign of collateral blood flow on CTA in patients with occlusion of the internal carotid artery or proximal middle cerebral artery (6). In our analysis, we observed that good DSA collaterals were associated with a higher CBV index in patients with AIS in the late therapeutic window when treated with EVT. A recent study reported that the CBV index was independently correlated with a favorable 90-day outcome in patients with acute basilar artery occlusion after EVT and that a low CBV index was associated with higher infarct growth rates (6, 8). In our adjusted multivariable logistic regression model, the CBV index was independently associated with the 90-day outcomes, which is a novel finding in this patient population. Thus, patients in the good functional outcome group had an increased ratio of average CBV in the hypoperfused brain or average CBV in the normal brain compared with patients with poor functional outcomes. Emphasizing the importance of this finding will provide a basis for obtaining a software-derived measure of collateral status rather than calculating an imaging score. Our results further strengthened the correlation between collateral circulation and post-stroke outcomes after EVT.

Our results are consistent with an extensive body of literature that has demonstrated correlations between favorable collateral status and good imaging and neurological outcomes following AIS in early time windows (9–11). In our analysis of patients treated in the late therapeutic window, no significant connection was found between the collateral scales and baseline demographic characteristics, such as age and sex. The robustness of collateral perfusion is a reliable predictor of recanalization after intravenous or endovascular therapy for acute ischemic stroke. Previous studies have indicated a connection between post-IVT recanalization and good collateral circulation in individuals with LVO (12, 13). Studies on the treatment of LVO in the early window have suggested that EVT may be most effective for patients with good collateral status, including *post hoc* analyses of the Multicenter Randomized Clinical Trial of Endovascular Treatment for Acute Ischemic Stroke in the Netherlands (MR CLEAN) and SOLITAIRE FR With the Intention for Thrombectomy (SWIFT) trials (11, 14, 15). Our findings are consistent with those of the SWIFT trial, which showed that better collaterals were associated with a higher likelihood of successful revascularization and improved clinical outcomes in cases of AIS of the anterior circulation during the late therapeutic window.

The DAWN and DEFUSE 3 studies both utilized perfusion imaging to determine those with a positive imaging profile, and the benefits of EVT for ischemic stroke when performed in the early window were greatest when perfusion imaging criteria were used to select patients (1). Our study, along with several others, identified younger age and low NIHSS scores as independent predictors for favorable clinical outcomes at the 3-month follow-up (mRS 90 d of 0–2) (16). An early assessment of the severity and prognosis of a condition is essential for successful long-term treatment (17, 18). The clinical relevance of a high CBV index in patients undergoing EVT may provide prognostic information after treatment. It is important that patients and their families receive precise prognostic information to assist clinicians in their treatment (19, 20). In patients with a higher CBV index, a closer follow-up after hospital discharge and/or more intensive therapies to reduce disability are warranted. Commercial CTP software platforms can quickly calculate the CBV index and present the results, which can save time and facilitate faster decision-making.

This study has a few limitations. First, this study was retrospective and was conducted in a single center with a small sample size; all patients with AIS underwent EVT, thus limiting the generalizability of the results. Second, Tmax at a single time point cannot accurately predict the tissue fate of an individual patient because it is subject to changes owing to factors such as blood pressure and collateral flow. We recognize that the margin of error from the automated perfusion software may cause differences between the observed and predicted infarct volumes. Automated perfusion software is not available in all hospitals, limiting the applicability of the CBV Index.

## 5 Conclusion

The CBV index is an independent neuroimaging predictor of the 90 day clinical outcomes in AIS patients treated within the late therapeutic window. Patients with a high CBV index were more likely to have favorable outcomes after EVT within the late therapeutic window. In addition, a higher CBV index reflects improved blood flow and a favorable collateral status on DSA.

## Data availability statement

The original contributions presented in the study are included in the article/supplementary material, further inquiries can be directed to the corresponding author.

## Ethics statement

Ethical review and approval was not required for the study on human participants in accordance with the local legislation and institutional requirements. Written informed consent from the patients/participants or patients/participants' legal guardian/next of kin was not required to participate in this study in accordance with the national legislation and the institutional requirements.

## Author contributions

AS: Data curation, Formal analysis, Investigation, Writing—original draft, Writing—review & editing. SL: Conceptualization, Methodology, Writing—review & editing. LZ: Visualization, Supervision, Validation, Writing—review & editing. YC: Conceptualization, Investigation, Writing—review & editing. ZJ: Visualization, Supervision, Validation, Writing—review & editing. HS: Visualization, Supervision, Validation, Writing—review & editing.
